# Stability, bioavailability, and cellular antioxidant activity of piperine complexed with cyclic glucans

**DOI:** 10.1007/s10068-025-01884-1

**Published:** 2025-05-12

**Authors:** Solji Cho, Yunkyoung Jung, Shin-Joung Rho, Yong-Ro Kim

**Affiliations:** 1 Department of Biosystems Engineering, Seoul National University, Seoul, 08826 Republic of Korea; 2 Convergence Major in Global Smart Farm, Seoul National University, Seoul, 08826 Republic of Korea; 3 Center for Food and Bioconvergence, Seoul National University, Seoul, 08826 Republic of Korea; 4 Research Institute of Agriculture and Life Sciences, Seoul National University, Seoul, 08826 Republic of Korea

**Keywords:** Piperine, Cyclic glucan, Stability, Bioavailability, Cellular antioxidant activity

## Abstract

Piperine, the primary bioactive compound in black pepper, was complexed with cyclic glucans, including cycloamylose (CA), α-cyclodextrin, and 2-hydroxypropyl-β-cyclodextrin, to investigate the complexation effects on its solubility, stability, bioavailability, and cellular antioxidant activity (CAA). The formation of inclusion complex (IC) significantly improved the phase solubility of piperine and enhanced its stability under ultraviolet light, heat, and acidic conditions. Additionally, IC increased the retention rate of piperine after in vitro digestion. Permeability analysis using a Caco-2 cell monolayer showed that IC samples, particularly CAIC, reduced the efflux ratio compared to free piperine by decreasing the apparent permeability coefficient from apical-to-basolateral (P_app (B-A)_). Moreover, the enhanced cellular uptake capacity of piperine in IC contributed to a marked improvement in its CAA, CAIC showing the most pronounced effect. Therefore, inclusion complexation with cyclic glucans, especially CA, can be a practical strategy to overcome the lower solubility and bioavailability of free piperine in various industrial applications.

**Supplementary Information:**

The online version contains supplementary material available at 10.1007/s10068-025-01884-1.

## Introduction

Piperine, the primary bioactive compound found in black pepper, exhibits various functional properties, including antioxidant, antimicrobial, anti-inflammatory, and anticancer activities (Haq et al., 2021; Smilkov et al., 2019). Additionally, piperine acts as a bioenhancer by improving the bioavailability and absorption of other functional drugs and supplements. It achieves this by enhancing the ultrastructure of intestinal microvilli and increasing nutrient absorption in the gastrointestinal system (Khatri and Awasthi, 2016). Previous studies have indicated that piperine increases the absorption of curcumin by enhancing intestinal absorption and inhibiting p-glycoprotein (p-gp), which is highly expressed in cancer cells (Singh et al., 2013). Moreover, recent innovative research suggests that black pepper extracts containing piperine may regulate the proliferation of virus particles in the human body, presenting a potential defense against COVID-19 (Choudhary et al., 2020). Despite its broad range of biological activities, the industrial applications of piperine remain limited due to several challenges. First, its low water solubility and dissolution significantly hinder its pharmacological efficacy and bioavailability (Smilkov et al., 2019). Second, piperine exhibits toxic effects at higher concentrations, further limiting its applicability (Pachauri et al., 2015). Lastly. piperine is structurally unstable and prone to degradation under environmental stresses such as ultraviolet (UV) light exposure, high temperatures, and acidic conditions (Chen et al., 2020).

To address these limitations and improve the bioavailability and bioactive potential of piperine, previous studies have explored strategies to enhance its solubility and stability. Various encapsulation systems, such as self-emulsifying drug delivery system, chitosan-coated liposomes, core–shell nanoparticles, zein nanoparticles, and inclusion complexes (IC), have been investigated for this purpose (Baspinar et al., 2018; Imam et al., 2021; Rezaei et al., 2019; Shao et al., 2015). Among these approaches, IC formed using cyclodextrins (CD) are widely used delivery systems (Sharma and Baldi, 2016). CD possess a hydrophobic cavity and a hydrophilic exterior, which enhance the solubility and stability of poorly soluble functional compounds, enabling their application in various industries. For example, β-CD has been shown to form IC with piperine at specific molar ratios, thereby improving its solubility and stability (Ezawa et al., 2019). Furthermore, nanoencapsulation of black pepper oleoresin containing piperine with hydroxypropyl β-cyclodextrin (HPCD) has also been shown to enhance its antioxidant and antibacterial activities (Teixeira et al., 2013). Besides conventional CD, which are cyclic glucans characterized by an annular structure with α–1,4 glycosidic bonds, cycloamylose (CA) can be used as a potential complexing agent for piperine due to its diverse cavity geometry and high solubility (> 100 g/100 mL) (Table 1). CA has a mechanism similar to CD, but has a larger hydrophobic cavity and a flexible structure compared to CD, affording it the advantage of being able to form complexes with bioactive substances of various sizes (Rho and Kim, 2022; Takemasa et al., 2020). Studies have reported that CA provides superior protection for guest molecules compared to β-CD, owing to its structural advantages (Lee et al., 2022; Park et al., 2019; Rho and Kim, 2022). For instance, CA has been shown to enhance the stability and bioavailability of fat-soluble vitamins and licorice extract by forming stable ICs and providing a physicochemical barrier (Lee et al., 2022; Rho and Kim, 2022).

**Table 1:** Chemical structure, molecular weight, and solubility of cyclic glucans and piperine

	Cycloamylose (CA)	α-Cyclodextrin (αCD)	2-Hydroxypropyl-β-cyclodextrin (HPCD)	Piperine
Structure				
Molecular weight (Da)	Av. 6,600	972.84	1431–1806	285.34
Solubility in water (g/100 mL)	> 100 (at 25 °C)^a^	14.5 (at 25 °C)	≥ 100 (at 25 °C)	0.004 (at 18 °C)^b^

^a^The solubility of CA in water at 25 °C was referenced in a previous study conducted by Larsen (2002)

^b^The solubility of piperine in water at 18 °C was referenced in a previous study by Chong et al. (2020)

For the application of functional materials in the food industry, factors such as exposure to UV or high temperatures during food processing and distribution must be taken into account (Farkas, 2006). Moreover, the stability and absorption rate of a functional substance in the body after oral intake are essential considerations (Toutain and Bousquet-mélou, 2004). Therefore, this study aimed to investigate the bioavailability and cellular antioxidant activity (CAA) of piperine by forming IC with CA and comparing their performance with αCD and HPCD with different cavity sizes and solubilities. To achieve this goal, IC formation was confirmed using scanning electron microscopy (SEM), powder X-ray diffraction (PXRD), differential scanning calorimetry (DSC), and fluorescence measurements. Moreover, the bioavailability of piperine was evaluated using a simulated GIT model and Caco-2 cell permeability assays, while its antioxidant capacity was assessed in Caco-2 cells. The results of this study provide valuable information on applicability of the cyclic glucan complexes as effective delivery systems for hydrophobic bioactive compounds.

## Materials and methods

### Materials

Piperine (purity ≥ 97%), α-cyclodextrin (αCD, purity ≥ 98%), 2′,7′-dichlorodi-hydrofluorescein diacetate (DCFH-DA), and 2,2′-azobis(2-amidinopropane) dihydrochloride (ABAP) were obtained from Sigma-Aldrich (St. Louis, MO, USA). Hydroxypropyl-β-cyclodextrin (HPCD, CAS No. 128446-35-5, purity ≥ 97%) was purchased from Zibo Qianhui Biological Technology Co., Ltd. (Shandong, China). Cycloamylose (CA) with an average molecular weight of 6600 Da (Lot No. GSR1020) was sourced from Ezaki Glico Co., Ltd. (Osaka, Japan). The human colon adenocarcinoma cell line Caco-2 (KCLB No. 30037.1, lot No. 45687) was provided by the Korean cell line bank (Seoul, Korea). Dulbecco’s modified Eagle’s medium (DMEM), containing 4500 mg/L D-glucose and L-glutamine, and Dulbecco’s phosphate buffered saline (DPBS) without calcium chloride and magnesium chloride were purchased from Welgene Inc. (Daegu, Korea). The EZ-Cytox enhanced cell viability assay kit, used for the WST (water-soluble tetrazolium salt) assay, was purchased from DoGen Bio Co., Ltd. (Seoul, Korea). All chemicals and reagents used were of analytical grade.

### Phase solubility analysis

Phase solubility tests were performed following the method described by Higuchi and Connors (1965), with slight modifications. Cyclic glucans were dissolved in distilled water at concentrations ranging from 1 to 30 mM for HPCD and 1 to 40 mM for CA and αCD. An excess amount of piperine (5 mM), dissolved in 99.9% ethanol (w/v), was added to cyclic glucan solutions (CA, αCD, and HPCD) at varying concentrations. The resulting phase suspensions were agitated in a shaking water bath (BS31, Jeio Tech Co., Ltd., Seoul, Korea) at 25 °C for 24 h. Subsequently, undissolved piperine was removed by filtration, and the quantity of piperine in the IC was analyzed using a UV/Vis spectrophotometer (UV-1650 PC, Shimadzu, Kyoto, Japan) at a wavelength of 343 nm.

### Preparation of inclusion complexes and physical mixture

Inclusion complexes (IC) with cyclic glucans were prepared based on a modified method previously used for fat-soluble vitamins (Rho and Kim, 2022). Piperine was dissolved in 99.9% ethanol at a concentration of 5 mM and mixed with cyclic glucan solutions (CA, αCD, and HPCD) dissolved in distilled water, resulting in a final ethanol concentration of 5% (v/v). The mixtures were incubated at 25 °C for 24 h to allow complexation. Undissolved piperine was removed, and the clear IC solutions were lyophilized to obtain IC powders. For comparison, physical mixtures (PM) were prepared by blending piperine and cyclic glucans in the same weight ratio (w/w) as used in the solid IC preparations.

### Characterization of inclusion complexes

The fluorescence excitation and emission spectra were measured using a fluorescence microplate reader (SpectraMax i3x Multi-mode microplate reader, Molecular devices, Sunnyvale, CA, USA). The concentration of cyclic glucans was gradually increased from 0 to 40 mM, while the final concentration of piperine was maintained at 0.2 mM across all samples. Fluorescence spectra of piperine in 5% ethanol (w/v) were recorded within an emission wavelength range of 380–600 nm in 2 nm increments, with an excitation wavelength of 340 nm.

The surface morphology of the samples was analyzed using field-emission scanning electron microscopy (FE-SEM, SIGMA, Carl Zeiss, Germany). Thin layers of each sample were deposited on copper double-sided tape and coated with a platinum layer for 120 s at a current of 30 mA before imaging.

Thermal properties of the samples were evaluated using a differential scanning calorimeter (Perkin-Elmer DSC 4000, Waltham, MA, USA). Approx. 5 mg of each sample was sealed in an aluminum pan, and thermograms were recorded over a temperature range of 30–200 °C at a scanning rate of 10 °C/min under a nitrogen carrier gas flow rate of 40 mL/min.

The X-ray diffraction patterns of the samples were obtained using a powder X-ray diffractometer (PXRD, D5005, Bruker AXS GmbH, Karlsruhe, Germany) equipped with CuKα radiation (λ = 1.5418 Å). The samples were scanned in the 2*θ* angle range of 3–40°, with a scan step size of 0.02° and a scan speed of 0.5 s/step.

### Stability study

The UV stability of piperine encapsulated within cyclic glucans was assessed as in previous research methods (Rho and Kim, 2022), with slight modification. Samples were exposed to UV irradiation in a UV chamber (40 × 35 × 27 cm) equipped with a G8T5 UVA lamp (Philips, Amsterdam, The Netherlands) and collected at specified time intervals over a 120 min period. Thermal stability was assessed under forced degradation conditions in accordance with guidelines provided by the ICH and FDA (Ngwa, 2010). Samples were incubated in a thermostatic water bath at 80 °C for durations of 1, 3, and 5 days. To evaluate pH stability, samples were adjusted to a pH of 2 using 1 N HCl and incubated for 2 h in a water bath at 37 °C under constant stirring. For all stability tests, the residual piperine content after exposure to UV light, heat, and acidic conditions was quantified using a UV/Vis spectrophotometer.

### In vitro digestion

The retention rate of piperine in the samples after in vitro digestion was determined using a gastrointestinal tract (GIT) model that simulates the conditions of the mouth, stomach, and small intestine. This procedure was conducted with slight modifications to the method reported in previous studies (Sarkar et al., 2009; Zhang et al., 2015). The final digesta, which passed through the simulated mouth, stomach, and small intestine phases, was centrifuged at 12,000 rpm for 5 min. The resulting supernatant was extracted using 99.9% ethanol, and the piperine concentration in the digested samples was quantified using a UV/Vis spectrophotometer at a wavelength of 343 nm. The retention rate (%) of piperine after in vitro digestion was calculated using the following Eq. (1):

1 $$\text{Retention rate} \left( \% \right) = {\frac{\text{Concentration of piperine after digestion}}{{\text{Concentration of piperine before digestion}}}} \times 100$$

### Viability assay

Samples treated in in Caco-2 cells for 24 h were incubated with a WST solution mixed with SF media (1:10, v/v) and subsequently incubated at 37 °C in a CO_2_ incubator (BF-40 CI, Biofree, Seoul, Korea) for 1 h. After incubation, the absorbance of the samples was measured at 450 nm using a microplate reader (Multiskan FC, Thermo Fisher Scientific, Inc., Waltham, MA, USA). Cell viability (%) was calculated by comparing the absorbance of the treated sample (Abs_sample_) with the absorbance of cells exposed to SF media alone (Abs_control_) using the following Eq. (2):

2 $$\text{Cell viability} \left( \% \right) = {\frac{{\text{Abs}_{\text{sample}} }}{{\text{Abs}_{\text{control}} }}} \times 100$$

### Transepithelial transport assay

Transport assays were conducted at concentrations non-toxic to cells, as determined by the cell viability test. To confirm the integrity of the Caco-2 cell monolayer, transepithelial electrical resistance (TEER) values of Caco-2 cells (9.0 × 10^4^ cells/well) were measured using an epithelial volt-ohmmeter equipped with 4 mm STX2 chopstick electrodes (EVOM2, World Precision Instruments, Sarasota, FL, USA). The TEER value was calculated using the following Eqs. (3) and (4):

3 $$\text{R}_{\text{TISSUE}} \left( \Omega \right) = \text{R}_{\text{TOTAL}} \left( \Omega \right) - \text{R}_{\text{BLANK}} \left( \Omega \right)$$

4 $$\text{TEER}_{\text{TISSUE}} \left( {\Omega \times \text{cm}^{2} } \right) = \text{R}_{\text{TISSUE}} \left( \Omega \right) \times \text{A}_{\text{MEMBRAME}} \left( {\text{cm}^{2} } \right)$$

where R_TISSUE_ is the resistance of the specific cell, R_TOTAL_ represents the resistance obtained from the cell monolayers on the semipermeable membrane, R_BLANK_ is the resistance of the semipermeable membrane without cells, TEER_TISSUE_ is the TEER value, and A_MEMBRANE_ denotes the membrane area.

The transepithelial transport assay was performed with a TEER value greater than 300 Ω cm^2^. TEER values were measured before and after the experiment. For the apical-to-basolateral (A-B) transport experiment, the Caco-2 monolayer was first measured for TEER, then gently shaken and rinsed with preheated HBSS (pH 7.4) for 20 min. Subsequently, 1.5 mL of the sample diluted in HBSS (pH 6.5) was added to the apical side, and 2.2 mL of HBSS (pH 7.4) was added to the basolateral side. The plates were incubated in a shaking incubator (SHI1, LABTron, Seoul, Korea) at 37 °C for 2 and 4 h, followed by measuring the piperine concentration on the basolateral side. For the basolateral-to-apical (B-A) transport experiment, the same procedure was followed, except the sample was applied to the basolateral side, and only HBSS (pH 6.5) was added to the apical side. The remaining piperine on the apical side was measured. The apparent permeability coefficient (P_app_, cm s^−1^) was calculated using the following Eq. (5):

5 $$\text{P}_{\text{app}} = {\frac{Q}{{A \times C_{0}} \times t}}$$

where *Q* is the total amount of sample permeated (mg), *A* is the diffusion area of the monolayer (cm^2^), *C*_*o*_ is the initial concentration of the sample (mg/mL), and *t* is the total duration of the experiments (s). The difference between P_app (B-A)_ and P_app (A-B)_ was used to define the efflux ratio (Zeng et al., 2017). The comparable efflux ratios were calculated using the ratio P_app (B-A)_/P_app (A-B)_.

### Cellular antioxidant activity

The CAA assay was conducted with slight modifications to the method suggested by Wolfe and Liu (2007). IC samples, diluted to contain 100 μM piperine, and free piperine dissolved in 1% DMSO were added to Caco-2 cells (2.5 × 10^4^ cells/well) cultured in a 96-well microplate for 24 h, followed by an additional 24 h incubation. All wells were washed with DPBS and treated with 25 μM DCFH-DA dissolved in serum-free media for 1 h. Subsequently, the cells were washed again with DPBS and treated with 600 μM ABAP dissolved in HBSS. The fluorescent intensity of 2′,7′-dichlorofluorescein (DCF) was measured using a fluorescence microplate reader with excitation at 485 nm and emission at 535 nm. CAA values were calculated using the following Eq. (6):

6 $$\text{CAA unit} = 100 - \left( {\frac{\smallint \text{SA}}{{\smallint \text{CA}}}} \right) \times 100$$

where ∫SA is the integrated area under the sample fluorescence intensity versus time curve, and ∫CA is the integrated area under the control curve.

### Statistical analysis

All experiments were conducted in triplicate, and the data are expressed as mean ± standard deviation (SD). Statistical significance was evaluated using IBM SPSS statistics for Windows, version 23.0 (IBM Corp., Armonk, NY, USA). A one-way ANOVA followed by Duncan’s multiple range test was used to determine differences between groups. A *p*-value of less than 0.05 was considered statistically significant.

## Results and discussion

### Phase solubility study

**Fig. 1:**
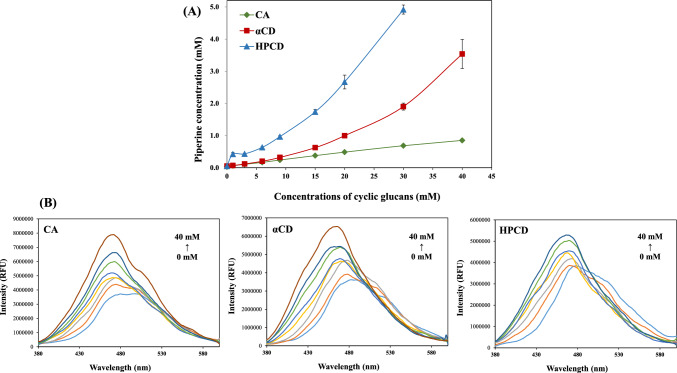
**A** Phase solubility diagrams and **B** fluorescence spectra of piperine at varying concentrations of cyclic glucans (CA, αCD, and HPCD). CA cycloamylose, αCD α-cyclodextrin, HPCD hydroxypropyl-β-cyclodextrin

Figure 1A shows the phase solubility diagrams for the IC formed between piperine and cyclic glucans. The solubility of free piperine in a 5% ethanol solution was extremely low, approx. 0.055 mM, consistent with previous study (Chong et al., 2020). This limited solubility can be attributed to the inherent hydrophobicity of piperine, which restricts its interaction with polar solvent. However, upon the addition of cyclic glucans, the solubility of piperine exhibited a significant concentration-dependent enhancement. The solubility enhancement was observed in the order of CA < αCD < HPCD, indicating that the structural characteristics, cavity size, and hydrophobicity of the cyclic glucans significantly influence their solubilization ability. Cyclic glucans, with their hydrophobic cavities, likely engage in molecular interactions such as hydrophobic interactions and hydrogen bonding with piperine, stabilizing its solubilized state (Jacob et al., 1998; Li et al., 2014; Misiuk and Zalewska, 2009). Quantitatively, the solubility of piperine increased approx. 12.6-fold, 34.6-fold, and 89.5-fold in the presence of 30 mM of CA, αCD, and HPCD, respectively. Previous studies have reported that both the methylenedioxy-phenyl group and the piperidine ring of piperine can be embedded within the hydrophobic cavity of CD, although the methylenedioxy-phenyl ring exhibits a higher occupancy rate (Ali et al., 2025; Ezawa et al., 2018). Therefore, this substantial increase observed was indicative of the effective formation of IC, wherein the hydrophobic moieties of piperine were encapsulated within the cyclic glucan cavities.

### Physicochemical characteristics of IC

#### Fluorescence spectroscopy measurements

The ability to form IC was confirmed by observing the change in fluorescence intensity of piperine with increasing concentration of cyclic glucans (Fig. 1B). In its free form, piperine emitted weak fluorescence at 480 nm, with intensity increasing as the concentration of cyclic glucans rose. This enhancement in fluorescence was particularly pronounced at 40 mM of cyclic glucans, where the fluorescence intensity was increased by 2.2-fold, 1.8-fold, and 1.4-fold for CA, αCD, and HPCD, respectively. Additionally, a slight blue shift of 10–15 nm in the emission spectra was observed in the presence of cyclic glucans, indicating changes in the environment around the piperine molecules. This shift and the intensity enhancement are indicative of the formation of IC. The hydrophobic cavities within the cyclic glucans are likely responsible for encapsulating the piperine molecules, preventing them from undergoing fluorescence quenching in the aqueous solution. The encapsulation shields the piperine from the surrounding water molecules, thereby stabilizing its fluorescence emission (Granadero et al., 2010).

#### Field emission-scanning electron microscopy

**Fig. 2:**
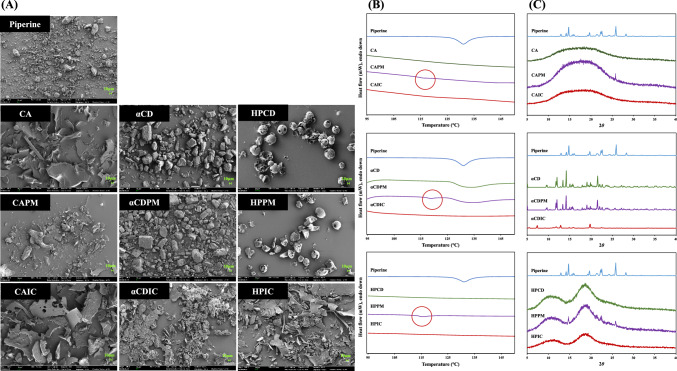
**A** The SEM images, **B** DSC thermograms, and **C** XRD patterns of piperine (free form), cyclic glucans, and their respective physical mixtures (PM) and inclusion complexes (IC). SEM images were measured at a magnification of 500 ×

The surface morphologies of free piperine, cyclic glucans (CA, αCD, and HPCD), as well as their PM and IC were analyzed using FE-SEM (Fig. 2A). CA exhibited a flat and smooth surface, whereas HPCD exhibited an amorphous spherical shape. The particles of αCD were relatively larger than those of piperine, and both showed irregular, fragmented shapes. In the case of PM, the individual components retained their intrinsic shapes, indicating no significant interaction between piperine and cyclic glucans. However, when examining IC, distinct morphological changes were observed. The particles in the IC samples displayed a unique, altered morphology, differing significantly from the individual components in the PM. Such structural alterations are consistent with previous studies, which have reported that the inclusion of guest molecules within the hydrophobic cavities of CD can lead to significant changes in the morphology of the resulting complexes (Ismail et al., 2021; Rajamohan et al., 2008). These results support the formation of molecular interactions between piperine and cyclic glucans in the solid state. The morphological changes observed in the IC samples suggest that the cyclic glucans successfully encapsulate piperine molecules within their cavities, resulting in a transformation of the physical characteristics of the complex.

#### Differential scanning calorimetry

Among the cyclic glucans, only αCD had a broad endothermic peak around 134 °C, whereas CA and HPCD showed no noticeable peaks (Fig. 2B). Previous studies have reported that αCD has a large endothermic peak between 40 and 170C, whereas CA and HPCD have no remarkable thermal peaks (Ho et al., 2015; Ismail et al., 2021; Kim, 2020). Piperine displayed a sharp melting endotherm at around 131 °C in its DSC profile, a result consistent with a previous study (Zaini et al., 2020). The endothermic peak of piperine appeared with a slight shift in the thermogram of PM. However, in the DSC thermograms of IC, the characteristic piperine peak disappeared. Additionally, for the αCDIC, the endothermic peak corresponding to αCD was also disappeared after the encapsulation process. The modification or complete disappearance of these peaks in the IC samples suggests a strong molecular interaction between piperine and the cyclic glucans, which may indicate the formation of stable IC (Hădărugă et al., 2019).

#### X-ray diffractometry

The XRD pattern of free piperine showed characteristic sharp crystallinity peaks (Fig. 2C). Both CA and HPCD displayed amorphous patterns without significant peaks, consistent with their non-crystalline structure. The diffraction pattern of the PM retained the characteristic peaks of piperine, indicating that the crystalline nature of piperine remained largely unchanged. For αCD, which showed crystallinity peaks, the diffraction pattern of its PM (αCDPM) also revealed distinct piperine peaks, conforming the lack of significant interaction between piperine and αCD in the physical state. In contrast, the XRD patterns of the IC demonstrated the disappearance of piperine’s sharp peaks, suggesting that piperine was no longer in its crystalline form. This transformation suggests that piperine was encapsulated within the hydrophobic cavities of the cyclic glucans, leading to the formation of new solid complexes (Ismail et al., 2021). The observed amorphous state of the IC could be attributed to molecular interactions, such as hydrogen bonding or hydrophobic interaction, between piperine and the cyclic glucans.

### Stability enhancement effect of IC

#### UV stability

**Fig. 3:**
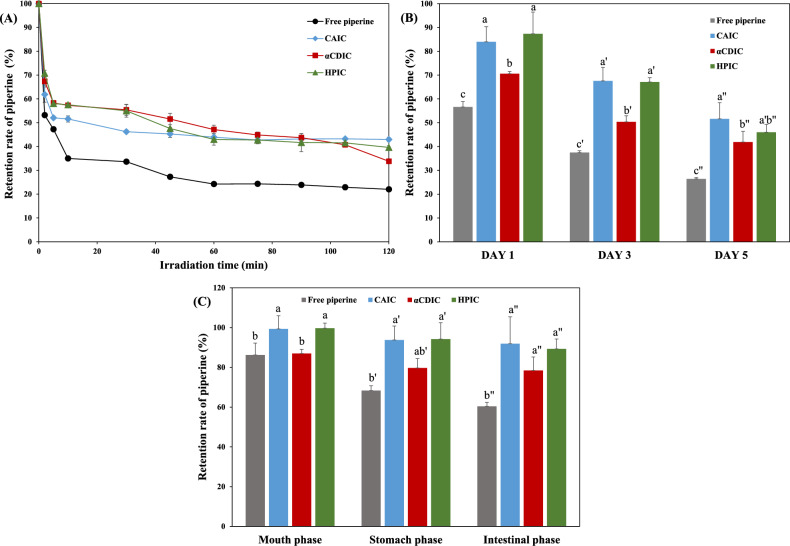
Changes in retention rate (%) of piperine in inclusion complexes (IC) prepared with cyclic glucans (CA, αCD, and HPCD) after **A** UV irradiation, **B** heat treatment at 80 °C, and **C** in vitro digestion via a simulated GIT model. Values represent the means ± standard deviation of triplicate experiments. Result marked with different letters above the bars indicate significant differences (*p* < 0.05)

Piperine, a compound with multiple conjugated double bonds and an aromatic ring, is highly susceptible to photodegradation when exposed to UV light (Guo et al., 2014; Tang et al., 2015). Structural instability under UV irradiation occurs due to processes such as chain scission, cross-linking, and substitutions in multiple bonds, ultimately leading to the formation of degradation products like trichostachine, iso-chavicin, and *cis*-piperylin (Kotte et al., 2014; Shaikh et al., 2006). Such degradation diminishes the bioactivity and stability of piperine, limiting its practical applications in food and pharmaceutical systems. Figure 3A presents the change in the retention rate (%) of piperine under UVA irradiation for 120 min. Free piperine exhibited a rapid and significant decline in the first 10 min of UVA exposure. In contrast, the IC demonstrated improved stability under identical conditions. Although piperine in IC also showed a decrease in retention during the initial 10 min, the degradation rate was slower than that of free piperine. Over time, the retention rate stabilized, with CAIC, αCDIC, and HPIC exhibiting significantly higher protection against photodegradation than free piperine by 1.95-, 1.53-, and 1.8-folds, respectively, after 120 min of UVA exposure. This enhanced stability can be attributed to the ability of cyclic glucans to encapsulate piperine within their hydrophobic cavities, shielding it from direct UV radiation and preventing photochemical reactions that lead to degradation.

#### Thermal stability

Changes in the thermal stability of piperine in response to complex formation with cyclic glucans are shown in Fig. 3B. Free piperine exhibited a gradual decline in retention during storage at 80 °C, with its content decreasing to approx. 26.4% after 5 days. This observation is consistent with previous studies that have demonstrated the susceptibility of piperine to thermal degradation and instability under high-temperature conditions (Chen et al., 2020). In contrast, the retention rates of piperine in all IC were significantly higher than those of free piperine under the same thermal stress. After 5 days of heat treatment, the thermal stability of piperine was improved by approx. 1.95-fold, 1.58-fold, and 1.74-fold in CAIC, αCDIC, and HPIC, respectively. These enhancements in thermal stability are consistent with findings from previous research. Rezaei et al. (2019) reported that complexation with CD improved the thermal stability of insoluble bioactive compounds, while Yildiz et al. (2018) observed similar protective effects menthol upon forming IC with HPCD. These studies, along with the current results, underscore the efficacy of cyclic glucans in protecting heat-sensitive bioactives like piperine from thermal degradation.

Interestingly, the trends observed in the UV and thermal stability (Fig. 3A and B) did not parallel the solubility trends (Fig. 1A). Despite CA demonstrating a lower solubility enhancement for piperine compared to HPCD or αCD at all concentrations, it provided UV and thermal stabilization effects similar to, or even better then, HPCD This discrepancy suggests that CA’s protective effects may stem from structural features rather than solely from its solubilizing capacity. CA, characterized by its DP ranging from 23 to 45, exhibits a unique cavity structure that likely contributes to its stabilizing effect. Unlike CD, which have a relatively shallow cavity formed by a single turn of glucan cyclization, cavity structure of CA can form helices with multiple turns of the glucose polymer, resulting in deeper and more versatile cavities (Ivanov et al., 2015). Molecular dynamics simulations have revealed that CA can adopt diverse conformations, such as double-stranded loops, parallel twisted helices, and symmetrical looped structures depending on its DP (Gotsev et al., 2007; Ivanov and Jaime, 2004; Ivanov et al., 2015). For example, CD26 has been described as a channel-like cavity structure composed of two short V-amylose helices oriented antiparallel (Nimz et al., 2004). Such variations in conformation provide CA with the ability to form deeper and more flexible cavities compared to traditional CDs, which are limited to a single shallow turn of glucan cyclization (Ismail et al., 2021; Kerdpol et al., 2021). The deeper cavity structure of CA likely enables it to encapsulate piperine more effectively, shielding it from external stresses such as heat and UV radiation. As previously discussed, both αCD and HPCD can enhance piperine solubility by forming inclusion complexes through two possible orientations—either via the methylenedioxy-phenyl ring or the piperidine ring (Ali et al., 2025; Ezawa et al., 2018). While this complexation contributes primarily to solubility enhancement, CA may offer additional structural benefits by stabilizing regions of the piperine molecule that are especially susceptible to degradation. In particular, the unique cavity structure of CA is expected to protect the conjugated double bond system of piperine, which is prone to UV- and heat-induced *cis–trans* isomerization. This protective effect may help prevent the conversion of *trans–trans* (piperine) form into its less stable isomeric forms, including *trans–cis* (iso-chavicin), *cis–cis* (carvicin), and *cis–trans* (iso-piperine) (Kozukue et al., 2007). Therefore, beyond its role in solubility enhancement, CA may confer superior stability to piperine by mitigating structural isomerization under thermal or photochemical stress.

#### Stability under acidic conditions

The protective effect of IC under acidic conditions was evaluated by measuring the retention of undestroyed piperine at pH 2 while maintaining a constant temperature of 37 °C. As shown in Fig. S1, free piperine exhibited a retention rate of approx. 68%, indicating significant degradation. This aligns with previous studies, which have demonstrated that piperine undergoes hydrolysis in highly acidic environments, resulting in degradation products such as piperic acid and piperidine (Kotte et al., 2014; Tiwari et al., 2020). In contrast, the IC samples showed markedly improved piperine retention with minimal degradation under same conditions. Additionally, despite the potential for acid hydrolysis of cyclic glucans themselves, the formation of IC appears to reduce their hydrolysis rate. This stabilization effect has been previously attributed to the interaction between the cyclic glucans and their guest molecules, which alters the susceptibility of the glucans to acid-mediated degradation (Vaitkus et al., 2011). Overall, these results suggest that the formation of IC significantly enhances the acidic stability of piperine, offering a promising approach to maintaining its bioactivity in environments with low pH, such as the gastric system.

### In vitro digestion

Figure 3C shows the retention rate (%) of piperine during the simulated GIT digestion process, which includes the mouth, stomach, and small intestine phases. Piperine is prone to degradation throughout the digestive process due to exposure to varying pH conditions and enzymatic actions. Particularly in the stomach, its instability under highly acidic conditions leads to a substantial decrease in bioaccessibility (Kotte et al., 2014; Quilaqueo et al., 2019). As shown in Fig. 3C, free piperine exhibited retention rates of 86.2, 68.4, and 60.4% at the mouth, stomach, and small intestine phases, respectively. These results confirmed its rapid decomposition as it progresses through the digestive tract, especially in the stomach, where acidic conditions accelerate its degradation.

In contrast, all IC samples showed significantly improved retention rates across all phase of the GIT. While αCDIC exhibited retention rates comparable to free piperine in the mouth phase, it showed a slight increase in the stomach and small intestine phases. CAIC and HPIC showed exceptional protective effects, with retention rates exceeding 99% during the mouth and stomach phases. This indicates that CAIC and HPIC effectively stabilized piperine under harsh acidic conditions. In the intestinal phase, retention rates for CAIC, αCDIC, and HPIC were 92%, 78.4%, and 89.3%, respectively, demonstrating a consistent trend of enhanced piperine stability across the digestion process. Interestingly, CAIC and HPIC exhibited no significant differences in retention rates across all GIT phases (Fig. 3C), which corresponded with the trends observed in stability studies (Fig. 3A and B). HPCD may provide enhanced stability through a higher number of atomic interactions with piperine, as previously reported (Ali et al., 2025). In the case of CA, this may be attributed to more flexible and the deeper cavity structure encapsulating a broader region of the piperine molecule, which more effectively protect piperine from the various stresses in the GIT. Our previous study also demonstrated that CA provided a distinct advantage in the retention rate of licorice extract (glabridin) after simulated GIT digestion, despite a lower solubilizing effect than HPCD (Lee et al., 2022). In conclusion, these results suggest that cyclic glucans act as physical barriers, effectively protecting piperine from enzymatic degradation or pH-induced instability during digestion process. Nevertheless, further investigations are needed to fully elucidate the underlying mechanisms governing this stabilization effect.

### Caco-2 cell viability assay

The cytotoxicity assessment of piperine on Caco-2 cells was evaluated using the WST assay to establish the maximal non-toxic concentration suitable for subsequent transport experiments. As a control group, free piperine was dissolved in 1% DMSO at a concentration of 100 μM, while all IC samples were diluted to contain an equivalent piperine concentration. The results of the WST assay showed that cell viability decreased in a concentration-dependent manner (Fig. S2A). Even at a piperine concentration of 100 μM, the cell viability remained above 95%, ensuring the integrity of the Caco-2 cellular membrane over a 24 h period (Fig. S2B). Generally, a cell viability of ≥ 80% is considered non-toxic to cells (Wahlang et al., 2011). Based on these results, the optimal concentration of piperine selected for the subsequent transport experiment via Caco-2 cell monolayer and in vitro antioxidant activity measurements was determined to be 100 μM.

### Transepithelial transport assay

**Fig. 4:**
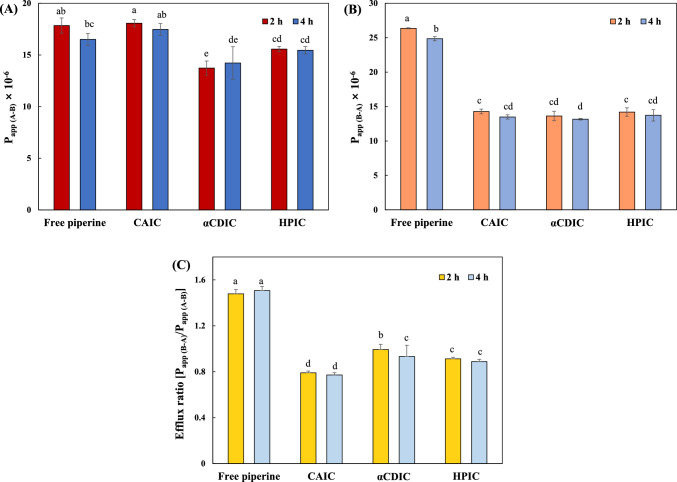
Apparent permeability coefficients (P_app_) of piperine on **A** the apical-to-basolateral (A-B) and **B** basolateral-to- apical (B-A) sides via Caco-2 cell monolayers for 2 h and 4 h. Values are reported in cm/s. **C** The efflux ratio, calculated as P_app (B-A)_/ P_app (A-B)_ at 2 h and 4 h. Result marked with different letters (a-d) above the bars indicate significant differences (*p* < 0.05)

Caco-2 cells, with properties resembling human intestinal cells, are widely utilized as an in vitro model to assess the absorption and transport mechanisms of orally administrated drugs across the intestinal epithelial layer. In particular, both active and passive transport processes can be examined by measuring permeability in the apical-to-basolateral (A-B) and basolateral-to-apical (B-A) directions (Hubatsch et al., 2007). In this study, the TEER values were maintained above 300 Ω × cm^2^ for the formation of Caco-2 cell monolayers. To evaluate the transport and absorption of IC and free piperine, permeability tests were performed over 0, 2, and 4 h to obtain the apparent permeability coefficients (P_app_) (Fig. 4). No significant differences in the piperine permeability were detected for the A-B or B-A sides of the Caco-2 cell monolayer with respect to permeation time (2 and 4 h). The P_app (A-B)_ values for CAIC were comparable to those of free piperine, while αCDIC and HPIC showed slightly reduced P_app (A-B)_ values (Fig. 4A). This suggests that while cyclic glucans enhance solubility of piperine, the improvement in solubility was not directly proportional to its intestinal absorption rate. A similar trend was observed in a study on curcumin, which found that HPCD enhanced solubility but did not significantly increase intestinal absorption (Li et al., 2018). These findings suggest that solubility enhancement by cyclic glucans does not necessarily translate to a proportional increase in absorption and that absorption mechanisms may vary depending on the drug’s molecular properties. Cyclic glucans, such as CDs, are known to facilitate the transport of hydrophobic drug molecules into cells via mechanisms like endocytosis, thereby improving the bioavailability of hydrophobic compounds (Réti-Nagy et al., 2015; Rusznyák et al., 2021, 2022).

The P_app (B-A)_ values of all IC were approx. half of those of free piperine (Fig. 4B), indicating that the cyclic glucans reduced piperine efflux in the B-A direction. Efflux ratio, calculated as the ratio of P_app (A-B)_ to P_app (B-A)_, revealed that free piperine had an efflux ratio of approx. 1.5 (Fig. 4C), which was similar to previous studies reporting substantial piperine efflux during membrane transport (Ren et al., 2018; Van Breemen and Li, 2005). In contrast, the efflux ratio for CAIC, αCDIC, and HPIC were significantly reduced to approx. 0.8, 0.9, and 0.9, respectively. This reduction suggests that cyclic glucans effectively modulate piperine’s transport by decreasing its during membrane crossing. Notably, CAIC showed the most substantial reduction in efflux, aligning with its superior performance in stabilizing piperine under various stress conditions (UV, heat, acidic pH, and digestion). Possible mechanisms by which CD complexes reduce the efflux of functional compounds include the inhibition of efflux transporters, modulation of tight junctions in the paracellular route, and alterations in membrane fluidity of lipid bilayer (Arima et al., 2001; Fenyvesi et al., 2011; Li et al., 2018). β-CD derivatives complexed with nintedanib (Vaidya et al., 2019) and tacrolimus (Arima et al., 2001) have been reported to reduce drug efflux across the membrane by inhibiting p-glycoprotein (p-gp) ATPase activity. In another study, Li et al. (2018) suggested that α-CD could transiently redistribute tight junction proteins in Caco-2 cell monolayers, thereby facilitating paracellular permeation of curcumin and reducing its exposure to efflux mechanisms. Another possible mechanism of efflux reduction could be that CD, a membrane cholesterol depleting agent, alter the overall packing of membrane lipids (Fenyvesi et al., 2011). CD derivatives extract cholesterol from the membrane, thereby altering the lipid raft domains where efflux transporters reside, thereby increasing membrane fluidity (Liu et al., 2022). Although the precise mechanisms underlying these effects have not yet been fully elucidated, these findings suggest the potential of CD complexes to modulate key transport pathways. Studies specifically addressing the cellular uptake and internalization mechanisms of CA remain also limited, suggesting the need for further research into its direct synergistic effects and involvement in metabolic pathways.

### In vitro antioxidant activity

**Fig. 5:**
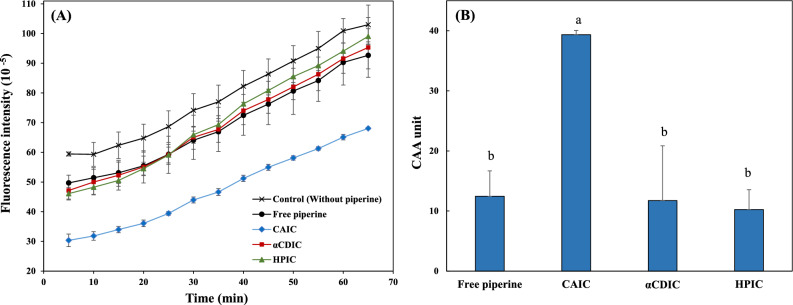
Cellular antioxidant activity (CAA) of piperine and inclusion complex (IC): **A** Kinetic curves of fluorescence from CAA assay and **B** comparison of CAA values between free piperine and piperine-loaded IC. Values represent means ± standard deviation of triplicate experiments. Result marked with different letters (a–b) above the bars indicate significant differences (*p* < 0.05)

The CAA of piperine was evaluated using an oxidative Caco-2 cell model. Peroxyl radicals generated by the addition of ABAP oxidize deacetylated DCFH from DCFH-DA after diffusion into Caco-2 cells. This oxidation produces DCF, a fluorescence compound whose intensity reflects intracellular oxidative stress (Wolfe and Liu, 2007). Substance with antioxidant activity inhibit this oxidation, resulting in reduced fluorescence intensity. The change in DCF fluorescence intensity of each sample over time is shown in Fig. 5A. The control group, without piperine, exhibited a continuous increase in DCF fluorescence intensity over time, indicating persistent production of DCF within the cells as oxidant ABAP was consumed. Free piperine and IC samples showed reduced DCF fluorescence compared to the control, suggesting their antioxidant potential. In particular, among the IC samples, CAIC showed significantly lower DCF fluorescence intensity compared to αCDIC, HPIC, and free piperine, indicating excellent antioxidant activity. When the CAA value was calculated based on the kinetic curves (Fig. 5B), the antioxidant capacity of CAIC was found to be approx. 3.2-fold higher than that of free piperine. This result suggests that encapsulation of piperine in CA substantially enhances its antioxidant activity in Caco-2 cells. The enhanced CAA of CAIC can be explained by several factors observed in this study. First, CAIC demonstrated superior protective capabilities for piperine against environmental stresses (UV, heat, acidic pH, and enzymatic digestion). This enhanced protection likely maintains a higher intracellular concentration of intact piperine within the Caco-2 cells, preserving its bioactivity and allowing it to exhibit stronger antioxidant effects. Second, the lower efflux ratio of CAIC compared to free piperine indicated that a larger amount of piperine was retained within the cells, further enhancing its antioxidant activity. The reduced efflux not only minimized the loss of piperine during transport across the cellular membrane but also increased its bioavailability within the cells. Previous studies have shown that improved cellular absorption of bioactive compounds, such as β-carotene encapsulated in micelles, can lead to significant enhancements in antioxidant activity (Du et al., 2019). Similarly, the unique structural properties of CA, particularly its deeper cavity and stronger encapsulation effect, appear to play a crucial role in facilitating piperine uptake by Caco-2 cells. This enhanced uptake likely contributed to the antioxidant capacity observed for CAIC in this study.

In conclusion, this study demonstrated that IC formation with cyclic glucans significantly enhances the solubility, stability, and bioavailability of piperine, while improving its CAA. The IC effectively protected piperine under stress conditions such as UV irradiation, high temperatures, and acidic environments by forming a protective physicochemical barrier. Enhanced piperine retention was also observed after a simulated gastrointestinal digestion process, suggesting the potential of cyclic glucans as carriers for oral delivery. IC samples, particularly CAIC, showed reduced efflux ratios and improved intestinal absorption, resulting in greater bioavailability. Additionally, CAIC exhibited a 3.2-fold higher CAA than the control, attributed to flexible structure and deeper cavities of CA, which provided superior environmental protection. These findings suggest the promise of cyclic glucans, especially CA, as innovative tools to develop more effective piperine-based formulations for food and pharmaceutical applications.

## Supplementary Information

Below is the link to the electronic supplementary material.

Supplementary file1 (DOCX 308 KB)

## References

[CR1] Ali S, Saokaew P, Aman A, Todsaporn D, Sanachai K, Krusong K, Hannongbua S, Wolschann P, Mahalapbutr P, Rungrotmongkol T. Enhancing solubility and stability of piperine using β-cyclodextrin derivatives: computational and experimental investigations. Journal of Biomolecular Structure and Dynamics. 43: 2596-2609 (2025) 38260962 10.1080/07391102.2024.2305696

[CR2] Arima H, Yunomae K, Hirayama F, Uekama K. Contribution of P-glycoprotein to the enhancing effects of dimethyl-β-cyclodextrin on oral bioavailability of tacrolimus. The Journal of Pharmacology and Experimental Therapeutics. 297: 547-555 (2001) 11303042

[CR3] Baspinar Y, Üstündas M, Bayraktar O, Sezgin C. Curcumin and piperine loaded zein-chitosan nanoparticles: development and in-vitro characterisation. Saudi Pharmaceutical Journal. 26: 323-334 (2018) 29556123 10.1016/j.jsps.2018.01.010 PMC5856953

[CR4] Chen S, Li Q, McClements DJ, Han Y, Dai L, Mao L, Gao Y. Co-delivery of curcumin and piperine in zein-carrageenan core-shell nanoparticles: Formation, structure, stability and in vitro gastrointestinal digestion. Food Hydrocolloids. 99: 105334 (2020)

[CR5] Chong WH, Chin SF, Pang SC, Kok KY. Synthesis and characterisation of piperine-loaded starch nanoparticles. Journal of Physical Science. 31: 57-68 (2020)

[CR6] Choudhary P, Chakdar H, Singh D, Selvaraj C, Singh SK, Kumar S, Saxena AK. Computational studies reveal piperine, the predominant oleoresin of black pepper (*Piper nigrum*) as a potential inhibitor of SARS-CoV-2 (COVID-19). Current Science. 119: 1333-1342 (2020)

[CR7] Du Y, Bao C, Huang J, Jiang P, Jiao L, Ren F, Li Y. Improved stability, epithelial permeability and cellular antioxidant activity of β-carotene via encapsulation by self-assembled α-lactalbumin micelles. Food Chemistry. 271: 707-714 (2019) 30236735 10.1016/j.foodchem.2018.07.216

[CR8] Ezawa T, Inoue Y, Murata I, Takao K, Sugita Y, Kanamoto I. Characterization of the dissolution behavior of piperine/cyclodextrins inclusion complexes. AAPS PharmSciTech. 19: 923-933 (2018) 29071656 10.1208/s12249-017-0908-9

[CR9] Ezawa T, Inoue Y, Murata I, Takao K, Sugita Y, Kanamoto I. Evaluation of the molecular state of piperine in cyclodextrin complexes by near-infrared spectroscopy and solid-state fluorescence measurements. International Journal of Medicinal Chemistry. 2019: 1-14 (2019) 10.1155/2019/7530480 PMC6388355 30886749

[CR10] Farkas J. Irradiation for better foods. Trends in Food Science & Technology. 17(4): 148-152 (2006)

[CR11] Fenyvesi F, Kiss T, Fenyvesi É, Szente L, Veszelka S, Deli MA, Váradi J, Fehér P, Ujhelyi Z, Tósaki Á, Vecsernyés M. Randomly methylated β‐cyclodextrin derivatives enhance taxol permeability through human intestinal epithelial Caco‐2 cell monolayer. Journal of Pharmaceutical Sciences. 100: 4734-4744 (2011) 21660974 10.1002/jps.22666

[CR12] Gotsev MG, Ivanov PM, Jaime C. Molecular dynamics study of the conformational dynamics and energetics of some large‐ring cyclodextrins (CDn, n = 24, 25, 26, 27, 28, 29). Chirality: The Pharmacological, Biological, and Chemical Consequences of Molecular Asymmetry. 19: 203-213 (2007) 10.1002/chir.20365 17226747

[CR13] Granadero D, Bordello J, Pérez-Alvite MJ, Novo M, Al-Soufi W. Host-guest complexation studied by fluorescence correlation spectroscopy: adamantane–cyclodextrin inclusion. International Journal of Molecular Sciences. 11: 173-188 (2010) 20162009 10.3390/ijms11010173 PMC2820997

[CR14] Guo L, Huang G, Zheng J, Li G. Thermal oxidative degradation of styrene-butadiene rubber (SBR) studied by 2D correlation analysis and kinetic analysis. Journal of Thermal Analysis and Calorimetry. 115: 647-657 (2014)

[CR15] Hădărugă NG, Bandur GN, David I, Hădărugă DI. A review on thermal analyses of cyclodextrins and cyclodextrin complexes. Environmental Chemistry Letters. 17: 349-373 (2019)

[CR16] Haq IU, Imran M, Nadeem M, Tufail T, Gondal TA, Mubarak MS. Piperine: a review of its biological effects. Phytotherapy Research. 35: 680-700 (2021) 32929825 10.1002/ptr.6855

[CR17] Higuchi T, Connors KA. Advances in analytical chemistry and instrumentation. Phase Solubility Studies. 4: 117-212 (1965)

[CR18] Ho TM, Howes T, Bhandari BR. Characterization of crystalline and spray-dried amorphous α-cyclodextrin powders. Powder Technology. 284: 585-594 (2015)

[CR19] Hubatsch I, Ragnarsson EG, Artursson P. Determination of drug permeability and prediction of drug absorption in Caco-2 monolayers. Nature Protocols. 2: 2111-2119 (2007) 17853866 10.1038/nprot.2007.303

[CR20] Imam SS, Alshehri S, Altamimi MA, Hussain A, Qamar W, Gilani SJ, Zafar A, Alruwaili NK, Alanazi S, Almutairy BK. Formulation of piperine–chitosan-coated liposomes: characterization and in vitro cytotoxic evaluation. Molecules. 26: 3281 (2021) 34072306 10.3390/molecules26113281 PMC8198173

[CR21] Ismail A, Kerdpol K, Rungrotmongkol T, Tananuwong K, Ueno T, Ekasit S, Muangsin N, Krusong K. Solubility enhancement of poorly water soluble domperidone by complexation with the large ring cyclodextrin. International Journal of Pharmaceutics. 606: 120909 (2021) 34298103 10.1016/j.ijpharm.2021.120909

[CR22] Ivanov PM, Jaime C. Insights into the structure of large-ring cyclodextrins through molecular dynamics simulations in solution. The Journal of Physical Chemistry B. 108: 6261-6274 (2004) 18950110 10.1021/jp037527t

[CR23] Ivanov PM, Atanassov EJ, Jaime C. Computational study on the intramolecular self-organization of the macrorings of some ‘giant’ cyclodextrins (CD n, n = 40, 70, 85, 100). Organic & Biomolecular Chemistry. 13: 1680-1689 (2015) 25465648 10.1039/c4ob02218a

[CR24] Jacob J, Geßler K, Hoffmann D, Sanbe H, Koizumi K, Smith SM, Takaha T, Saenger W. Strain‐induced “band flips” in cyclodecaamylose and higher homologues. Angewandte Chemie International Edition. 37: 605-609 (1998) 29711068 10.1002/(SICI)1521-3773(19980316)37:5<605::AID-ANIE605>3.0.CO;2-C

[CR25] Kerdpol K, Nutho B, Krusong K, Poo-arporn RP, Rungrotmongkol T, Hannongbua S. Encapsulation of α-tocopherol in large-ring cyclodextrin containing 26 α-D-glucopyranose units: a molecular dynamics study. Journal of Molecular Liquids. 339: 116802 (2021)

[CR26] Khatri S, Awasthi R. Piperine containing floating microspheres: an approach for drug targeting to the upper gastrointestinal tract. Drug Delivery and Translational Research. 6: 299-307 (2016) 26902907 10.1007/s13346-016-0285-z

[CR27] Kim JS. Study of flavonoid/hydroxypropyl-β-cyclodextrin inclusion complexes by UV-Vis, FT-IR, DSC, and X-ray diffraction analysis. Preventive Nutrition and Food Science. 25: 449 (2020) 33505939 10.3746/pnf.2020.25.4.449 PMC7813604

[CR28] Kotte SCB, Dubey PK, Murali PM. Identification and characterization of stress degradation products of piperine and profiling of a black pepper (*Piper nigrum* L.) extract using LC/Q-TOF-dual ESI-MS. Analytical Methods. 6: 8022-8029 (2014)

[CR29] Kozukue N, Park MS, Choi SH, Lee SU, Ohnishi-Kameyama M, Levin CE, Friedman M. Kinetics of light-induced cis–trans isomerization of four piperines and their levels in ground black peppers as determined by HPLC and LC/MS. Journal of Agricultural and Food Chemistry. 55: 7131–7139 (2007) 17661483 10.1021/jf070831p

[CR30] Larsen KL. Large cyclodextrins. Journal of Inclusion Phenomena and Macrocyclic Chemistry. 43: 1-13 (2002)

[CR31] Lee J, Jung Y, Rho SJ, Kim YR. Physicochemical characteristics and in vitro bioavailability of licorice (*Glycyrrhiza glabra* L.) extract complexed using cyclic glucans. LWT-Food Science and Technology. 167: 113841 (2022)

[CR32] Li Z, Chen S, Gu Z, Chen J, Wu J. Alpha-cyclodextrin: enzymatic production and food applications. Trends in Food Science & Technology. 35: 151-160 (2014)

[CR33] Li X, Uehara S, Sawangrat K, Morishita M, Kusamori K, Katsumi H, Sakane T, Yamamoto A. Improvement of intestinal absorption of curcumin by cyclodextrins and the mechanisms underlying absorption enhancement. International Journal of Pharmaceutics. 535: 340-349 (2018) 29157961 10.1016/j.ijpharm.2017.11.032

[CR34] Liu W, Han Y, Xin X, Chen L, Liu Y, Liu C, Zhang X, Jin M, Jin J, Gao Z, Huang W. Biomimetic and temporal-controlled nanocarriers with ileum transporter targeting for achieving oral administration of chemotherapeutic drugs. Journal of Nanobiotechnology. 20: 281 (2022) 35705976 10.1186/s12951-022-01460-3 PMC9199201

[CR35] Misiuk W, Zalewska M. Investigation of inclusion complex of trazodone hydrochloride with hydroxypropyl-β-cyclodextrin. Carbohydrate Polymers. 77: 482-488 (2009)

[CR36] Ngwa G. Forced degradation as an integral part of HPLC stability-indicating method development. Drug Delivery Technology. 10: 56-59 (2010)

[CR37] Nimz O, Gessler K, Usón I, Sheldrick GM, Saenger W. Inclusion complexes of V-amylose with undecanoic acid and dodecanol at atomic resolution: X-ray structures with cycloamylose containing 26 D-glucoses (cyclohexaicosaose) as host. Carbohydrate Research. 339: 1427-1437 (2004) 15178384 10.1016/j.carres.2004.02.030

[CR38] Pachauri M, Gupta ED, Ghosh PC. Piperine loaded PEG-PLGA nanoparticles: preparation, characterization and targeted delivery for adjuvant breast cancer chemotherapy. Journal of Drug Delivery Science and Technology. 29: 269-282 (2015)

[CR39] Park J, Rho SJ, Kim YR. Enhancing antioxidant and antimicrobial activity of carnosic acid in rosemary (*Rosmarinus officinalis* L.) extract by complexation with cyclic glucans. Food Chemistry. 299: 125119 (2019) 31295638 10.1016/j.foodchem.2019.125119

[CR40] Quilaqueo M, Millao S, Luzardo-Ocampo I, Campos-Vega R, Acevedo F, Shene C, Rubilar M. Inclusion of piperine in β-cyclodextrin complexes improves their bioaccessibility and in vitro antioxidant capacity. Food Hydrocolloids. 91: 143-152 (2019)

[CR41] Rajamohan R, Nayaki SK, Swaminathan M. Inclusion complexation and photoprototropic behaviour of 3-amino-5-nitrobenzisothiazole with β-cyclodextrin. Spectrochimica Acta Part A: Molecular and Biomolecular Spectroscopy. 69: 371-377 (2008) 17604682 10.1016/j.saa.2007.04.008

[CR42] Ren T, Wang Q, Li C, Yang M, Zuo Z. Efficient brain uptake of piperine and its pharmacokinetics characterization after oral administration. Xenobiotica. 48: 1249-1257 (2018) 29160763 10.1080/00498254.2017.1405293

[CR43] Réti-Nagy K, Malanga M, Fenyvesi É, Szente L, Vámosi G, Váradi J, Bácskay I, Fehér P, Ujhelyi Z, Róka E, Vecsernyés M, Balogh G, Vasvári G, Fenyvesi F. Endocytosis of fluorescent cyclodextrins by intestinal Caco-2 cells and its role in paclitaxel drug delivery. International Journal of Pharmaceutics. 496: 509-517 (2015) 26498369 10.1016/j.ijpharm.2015.10.049

[CR44] Rezaei A, Fathi M, Jafari SM. Nanoencapsulation of hydrophobic and low-soluble food bioactive compounds within different nanocarriers. Food Hydrocolloids. 88: 146-162 (2019)

[CR45] Rho SJ, Kim YR. Improving solubility and stability of fat-soluble vitamins (A, D, E, and K) using large-ring cycloamylose. LWT-Food Science and Technology. 153: 112502 (2022)

[CR46] Rusznyák Á, Malanga M, Fenyvesi É, Szente L, Váradi J, Bácskay I, Vecsernyés M, Vasvári G, Haimhoffer Á, Fehér P, Ujhelyi Z, Nagy Jr B, Fejes Z, Fenyvesi F. Investigation of the cellular effects of beta-cyclodextrin derivatives on Caco-2 intestinal epithelial cells. Pharmaceutics. 13: 157 (2021) 33504045 10.3390/pharmaceutics13020157 PMC7911713

[CR47] Rusznyák Á, Palicskó M, Malanga M, Fenyvesi É, Szente L, Váradi J, Bácskay I, Vecsernyés M, Réti-Nagy KS, Vasvári G, Haimhoffer Á, Fenyvesi F. Cellular effects of cyclodextrins: studies on HeLa cells. Molecules. 27: 1589 (2022) 35268690 10.3390/molecules27051589 PMC8911813

[CR48] Sarkar A, Goh KK, Singh H. Colloidal stability and interactions of milk-protein-stabilized emulsions in an artificial saliva. Food Hydrocolloids. 23: 1270-1278 (2009)

[CR49] Shaikh J, Bhosale R, Singhal R. Microencapsulation of black pepper oleoresin. Food Chemistry. 94: 105-110 (2006)

[CR50] Shao B, Cui C, Ji H, Tang J, Wang Z, Liu H, Qin M, Wu L. Enhanced oral bioavailability of piperine by self-emulsifying drug delivery systems: in vitro, in vivo and in situ intestinal permeability studies. Drug Delivery. 22: 740-747 (2015) 24670090 10.3109/10717544.2014.898109

[CR51] Sharma N, Baldi A. Exploring versatile applications of cyclodextrins: an overview. Drug Delivery. 23: 729-747 (2016) 10.3109/10717544.2014.938839 25051096

[CR52] Singh DV, Godbole MM, Misra K. A plausible explanation for enhanced bioavailability of P-gp substrates in presence of piperine: simulation for next generation of P-gp inhibitors. Journal of Molecular Modeling. 19: 227-238 (2013) 22864626 10.1007/s00894-012-1535-8

[CR53] Smilkov K, Ackova DG, Cvetkovski A, Ruskovska T, Vidovic B, Atalay, M. Piperine: old spice and new nutraceutical? Current Pharmaceutical Design. 25: 1729-1739 (2019) 31267856 10.2174/1381612825666190701150803

[CR54] Takemasa M, Yuguchi Y, Kitamura S. Size and shape of cycloamylose estimated using column chromatography coupled with small-angle X-ray scattering. Food Hydrocolloids. 108: 105948 (2020)

[CR55] Tang M, Xing W, Wu J, Huang G, Xiang K, Guo L, Li G. Graphene as a prominent antioxidant for diolefin elastomers. Journal of Materials Chemistry A. 3: 5942-5948 (2015)

[CR56] Teixeira BN, Ozdemir N, Hill LE, Gomes CL. Synthesis and characterization of nano‐encapsulated black pepper oleoresin using hydroxypropyl beta‐cyclodextrin for antioxidant and antimicrobial applications. Journal of Food Science. 78: N1913-N1920 (2013) 24329956 10.1111/1750-3841.12312

[CR57] Tiwari A, Mahadik KR, Gabhe SY. Piperine: a comprehensive review of methods of isolation, purification, and biological properties. Medicine in Drug Discovery. 7: 100027 (2020)

[CR58] Toutain PL, Bousquet‐mélou A. Bioavailability and its assessment. Journal of Veterinary Pharmacology and Therapeutics. 27: 455-466 (2004) 15601440 10.1111/j.1365-2885.2004.00604.x

[CR59] Vaidya B, Shukla SK, Kolluru S, Huen M, Mulla N, Mehra N, Kanabar D, Palakurthi S, Ayehunie S, Nuth A, Gupta V. Nintedanib-cyclodextrin complex to improve bio-activity and intestinal permeability. Carbohydrate Polymers. 204: 68-77 (2019) 30366544 10.1016/j.carbpol.2018.09.080

[CR60] Vaitkus R, Grinciene G, Norkus E. Inhibition of cyclodextrin acid hydrolysis by some inclusion complexes. Journal of Inclusion Phenomena and Macrocyclic Chemistry. 69: 345-347 (2011)

[CR61] Van Breemen RB, Li Y. Caco-2 cell permeability assays to measure drug absorption. Expert Opinion on Drug Metabolism & Toxicology. 1: 175-185 (2005) 16922635 10.1517/17425255.1.2.175

[CR62] Wahlang B, Pawar YB, Bansal AK. Identification of permeability-related hurdles in oral delivery of curcumin using the Caco-2 cell model. European Journal of Pharmaceutics and Biopharmaceutics. 77: 275-282 (2011) 21147222 10.1016/j.ejpb.2010.12.006

[CR63] Wolfe KL, Liu RH. Cellular antioxidant activity (CAA) assay for assessing antioxidants, foods, and dietary supplements. Journal of Agricultural and Food Chemistry. 55: 8896-8907 (2007) 17902627 10.1021/jf0715166

[CR64] Yildiz ZI, Celebioglu A, Kilic ME, Durgun E, Uyar T. Menthol/cyclodextrin inclusion complex nanofibers: Enhanced water-solubility and high-temperature stability of menthol. Journal of Food Engineering. 224: 27-36 (2018)

[CR65] Zaini E, Afriyani, Fitriani L, Ismed F, Horikawa A, Uekusa H. Improved solubility and dissolution rates in novel multicomponent crystals of piperine with succinic acid. Scientia Pharmaceutical. 88: 21 (2020)

[CR66] Zeng Z, Shen ZL, Zhai S, Xu JL, Liang H, Shen Q, Li QY. Transport of curcumin derivatives in Caco-2 cell monolayers. European Journal of Pharmaceutics and Biopharmaceutics. 117: 123-131 (2017) 28396278 10.1016/j.ejpb.2017.04.004

[CR67] Zhang R, Zhang Z, Zhang H, Decker EA, McClements DJ. Influence of emulsifier type on gastrointestinal fate of oil-in-water emulsions containing anionic dietary fiber (pectin). Food Hydrocolloids. 45: 175-185 (2015)

