# Supplementary material for: Stability, bioavailability, and cellular antioxidant activity of piperine complexed with cyclic glucans
Source: Food Sci Biotechnol. 2025 May 12;34(11):2475–88. doi: 10.1007/s10068-025-01884-1 (PMC12145342; doi:10.1007/s10068-025-01884-1)
Supplement: Supplementary file 1 — Supplementary file1 (DOCX 308 KB) [file 10068_2025_1884_MOESM1_ESM.docx]

**Supplementary data**


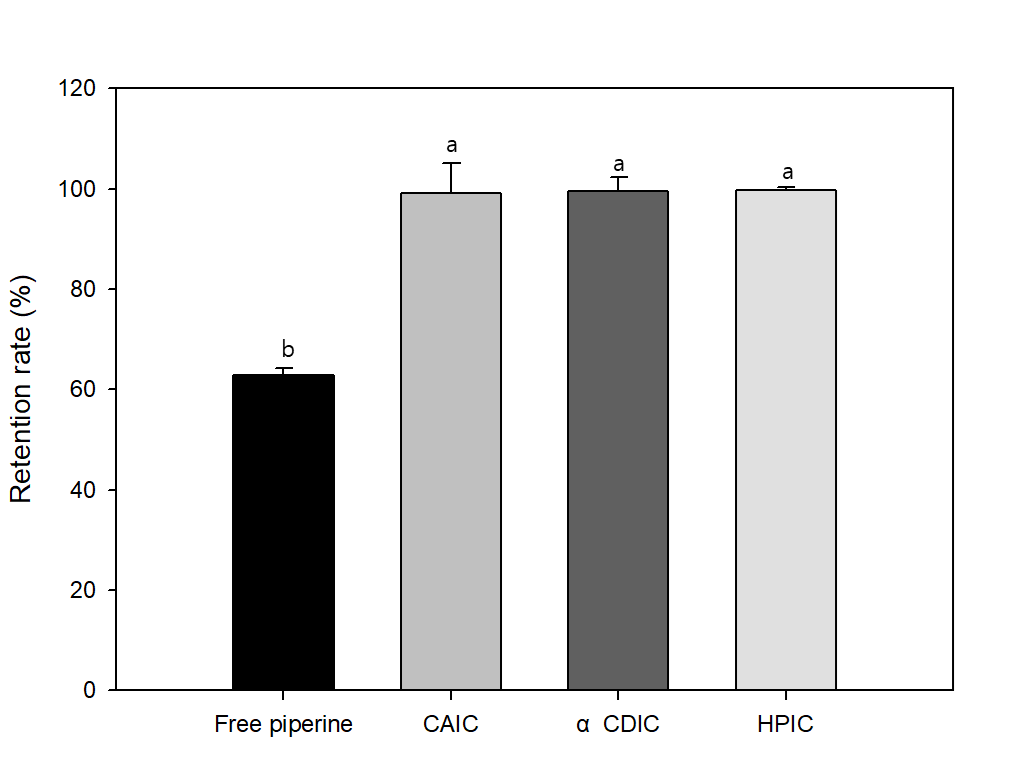


Figure S1. Changes in retention rate (%) of piperine in inclusion complexes (IC) prepared using cyclic glucans (CA, αCD, and HPCD) under acidic conditions (pH 2) at 37 ℃.


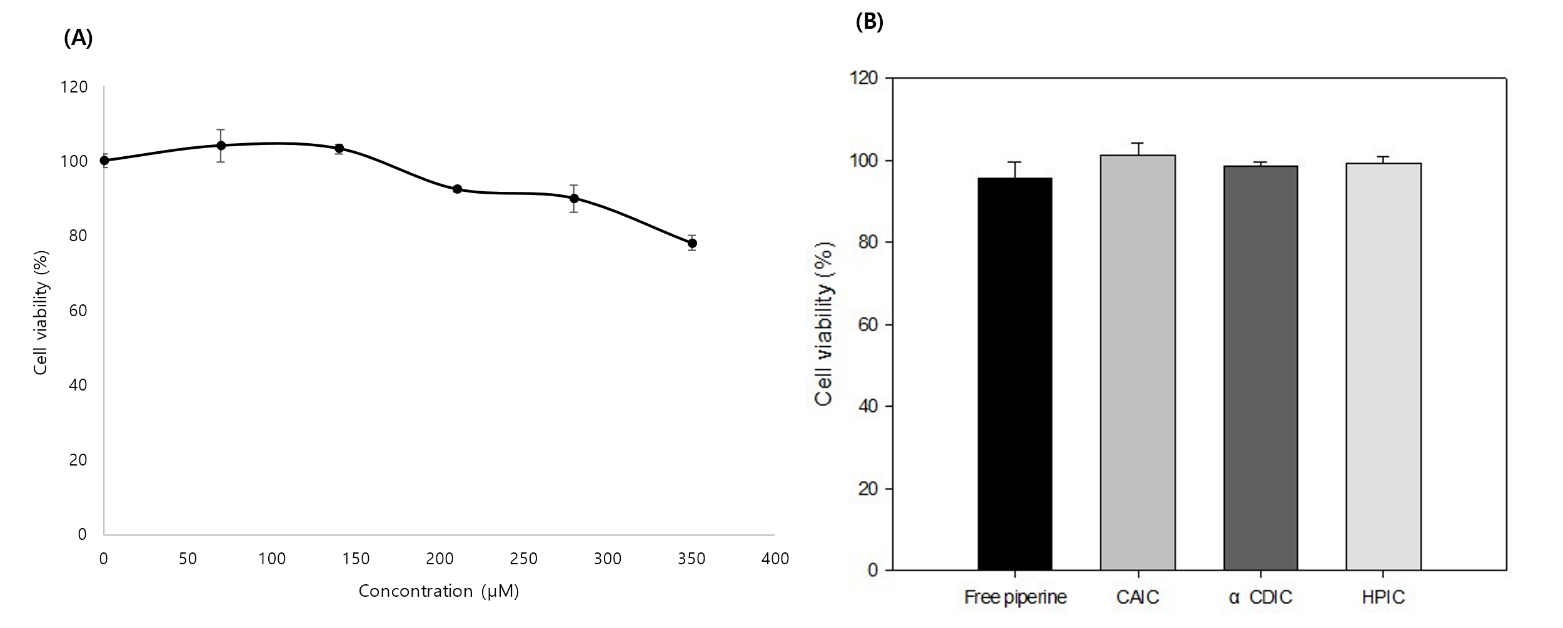


Figure S2. (A) Cell viability of Caco-2 cells treated with various concentrations of free piperine, evaluated using an MTT assay. (B) Changes in cell viability of samples containing 100 μM piperine in different forms, including free piperine and IC prepared with cyclic glucans (CA, αCD, and HPCD).
